# CT acquisition protocols in lung cancer screening: implications for guideline development from a worldwide survey

**DOI:** 10.1186/s13244-026-02239-y

**Published:** 2026-03-16

**Authors:** Mathis Franz Georg Konrad, Emily Nischwitz, Joanna Chorostowska-Wynimko, Anna Kerpel-Fronius, Viktoria Palm, Joanna Moes-Sosnowska, Mariusz Adamek, Gudrun Zahlmann, Aad van der Lugt, Jens Vogel-Claussen, Helmut Prosch, Hans-Ulrich Kauczor

**Affiliations:** 1 Department of Diagnostic and Interventional Radiology (DIR), Heidelberg University Hospital, Heidelberg, Germany; 2 Translational Lung Research Center Heidelberg (TLRC), German Center for Lung Research (DZL), Heidelberg, Germany; 3 Department of Genetics and Clinical Immunology, National Institute of Tuberculosis and Lung Diseases, Warsaw, Poland; 4 Department of Radiology, National Korányi Institute for Pulmonology, Budapest, Hungary; 5 Department of Diagnostic and Interventional Radiology with Nuclear Medicine, Thoraxklinik, Heidelberg University Hospital, Heidelberg, Germany; 6 Department of Thoracic Surgery, Medical University of Silesia, Katowice, Poland; 7 Department of Thoracic Surgery, Medical University of Gdańsk, Gdańsk, Poland; 8 Quantitative Medical Imaging Coalition, Ann Arbor, MI USA; 9 Department of Radiology and Nuclear Medicine, Erasmus MC - University Medical Center Rotterdam, Rotterdam, Netherlands; 10 Department of Diagnostic and Interventional Radiology, Hannover Medical School, Hannover, Germany; 11 German Center for Lung Research, Biomedical Research in Endstage and Obstructive Lung Disease (BREATH), Hannover Medical School, Hannover, Germany; 12 Department of Radiology, Charité Universitätsmedizin Berlin, Berlin, Germany; 13 Department of Biomedical Imaging and Image-guided Therapy, Medical University of Vienna, Vienna, Austria

**Keywords:** Tomography (X-ray computed), Mass screening, Thorax, Radiation exposure, Lung neoplasms

## Abstract

**Objectives:**

To assess the currently applied CT image acquisition protocols in lung cancer screening (LCS) and thereby fill a knowledge gap to support guideline development.

**Materials and methods:**

Through worldwide distribution of an online survey, data on institutional and technical factors regarding CT acquisition protocols in LCS were collected between 06/2024 and 09/2025 on behalf of the SOLACE (Strengthening the screening of lung cancer in Europe) consortium.

**Results:**

Global responses were received from 71 LCS institutions across 29 countries (all continents). Responsibility for CT protocol establishment and modification varied among professions (radiologists, radiographers, medical physicists, and manufacturer personnel). Protocol establishment was dominated by radiologists (64 of 115), with only one-third of institutions involving multiple professions. Technical questions were partially answered. Automatic exposure control was implemented in 88% of centers (43 of 49). Reconstructed slice thickness ranged from 0.625 to 1.5 mm, with 1.0 mm being most common (43 of 67). Increment ranged between 0.5 and 1.25 mm. Software support for LCS was used by 90% of respondents (35 of 39), primarily for nodule detection (92%), volumetry (89%), and calculation of volume doubling time (71%). Image reconstruction was dominated by iterative reconstruction with statistical modeling (30) or deep learning support (7), while filtered-back projection was marginally used (4).

**Conclusions:**

Lung cancer screening often pushes current device limits, which warrants a multiprofessional establishment of CT protocols. Variability in reconstruction calls for further study on the effects on volumetry. Optimizing protocols remains crucial to balance radiation dose reduction and diagnostic accuracy in guideline development.

**Critical relevance statement:**

This international study evaluates current CT image acquisition protocols in lung cancer screening and implications for guidelines, highlighting insufficient multiprofessional engagement for protocol definition and pronounced variability in technical parameters, both of which demand harmonization to inform robust guideline development.

**Key Points:**

- Variability of CT acquisition protocols impacts lung cancer screening.
- International survey results shed light on currently applied protocols.
- The narrowed knowledge gap supports guideline recommendations and standardization.

**Graphical Abstract:**

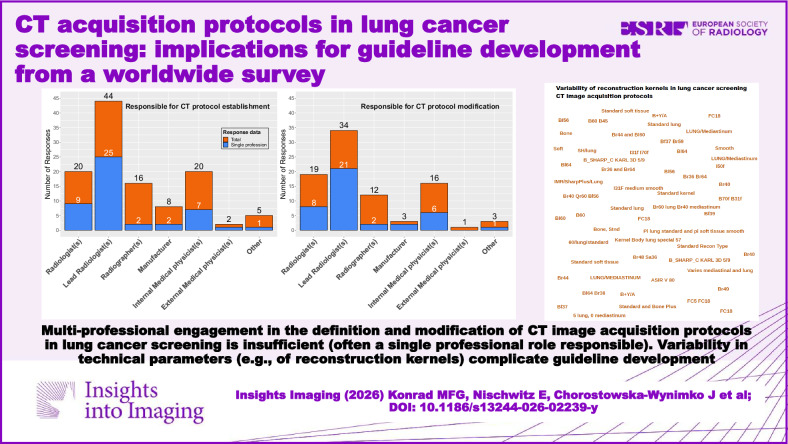

## Introduction

Lung cancer remains one of the most significant global health challenges, accounting for substantial morbidity and mortality worldwide [1]. Early detection through low-dose computed tomography (CT) screening has emerged as a promising approach to reduce lung cancer mortality [2], and its implementation is expanding across Europe and internationally [3]. However, considerable variation persists in CT acquisition protocols employed for lung cancer screening (LCS), presenting critical challenges for standardization, quality assurance (QA), and reliable quantification. To address these gaps, a comprehensive survey initiative was recently launched [4].

There is longstanding recognition that volumetric assessment of small pulmonary nodules detected in CT images enables determination of nodule growth rates, offering a sensitive method for early detection and management decisions [5]. More recent research has focused on the methodological dependencies of volumetric analysis, underlining the influence of differing software solutions on the measurement of nodule growth and highlighting the necessity for protocol and software standardization across institutions [6]. Consistent and reproducible nodule volume determination is indispensable for calculation of volume doubling time (VDT), a quantitative imaging biomarker central to contemporary guidelines for LCS nodule management [7].

Robust technical QA is highly relevant to ensure reliable and reproducible quantitative imaging biomarkers, particularly in the context of low-dose CT image acquisition used for LCS [8]. Advances in CT image reconstruction techniques, including the integration of deep learning-based image reconstruction and secondary analysis with AI algorithms, have demonstrated potential for dose reduction and enhanced diagnostic consistency in screening settings [9, 10]. Nevertheless, regulatory frameworks vary; for example, in Germany, the inclusion of software for nodule detection in LCS is a legal requirement [11], though the optimal role and performance of AI-based solutions remain under investigation [10].

Beyond clinical and technical considerations, the cost-effectiveness of LCS programs has been reinforced by recent economic analyses, with substantial financial savings linked to early lung cancer detection and prevention strategies [12]. As the paradigm continues to shift toward more comprehensive CT-based screening, addressing protocol heterogeneity becomes paramount to reliably extend screening benefits beyond lung cancer alone [13].

An ESR/ERS (European Society of Radiology/European Respiratory Society) statement paper emphasized the urgent need for consistent guideline development [14]. The Strengthening the Screening of Lung Cancer in Europe (SOLACE) consortium has taken up these efforts and is addressing cross-institutional standardization to maximize the impact and sustainability of LCS programs, thus further promoting equity and improving outcomes across diverse populations [3, 4].

## Materials and methods

This cross-sectional, electronic, voluntary network-based professional survey assessed institutional and technical practices in CT image acquisition for LCS. Institutional representatives involved in LCS protocols from SOLACE consortium networks and affiliated societies were invited to participate. The institution name was requested from responders to avoid duplicate entries. All institutions implementing LCS were eligible.

### Survey design

A two-page electronic survey was developed to collect information relevant to technical guideline recommendations and standardization of CT image acquisition protocols. The survey questions were developed by expert consensus (SOLACE Radiation Exposure Subgroup) and refined in collaboration with the European Imaging Biomarkers Alliance (EIBALL), the American Lung Association (ALA), the Small Lung Nodule Biomarker Committee of the Quantitative Medical Imaging Coalition (QMIC), formerly Quantitative Imaging Biomarkers Alliance (QIBA), and the European Society of Thoracic Imaging (ESTI); no formal pilot testing or Delphi-type validation was performed. The first page contained questions on general and institutional factors that incorporated select items from Demb et al [15]. The second page focused on technical factors. Partial survey responses were stored to enable maximum information yield and considered sufficient in the case that at least the general questions and questions regarding institutional factors were fully answered. The full survey is reported in Electronic Supplementary Material (ESM) Table 3.

### General questions

Survey participants were asked about the country in which the respective LCS center is located. In addition, they were queried as to whether LCS at their institution was performed using a single dedicated device, two devices, or more than two devices. Following the question regarding the number of devices dedicated to screening, participants were also asked to provide specific details on each device used, including the device name and manufacturer, via open text fields.

### Institutional factors

The survey questions covering institutional factors potentially influencing CT protocols included the responsibility of establishment and modification of protocols by personnel and the frequency of protocol updates. Separate answer options regarding the role of the individuals responsible for the establishment of the CT image acquisition protocols were: lead radiologist(s), radiologist(s), radiographer(s), manufacturer, internal medical physicist(s), external medical physicist(s), and other. An additional survey question asked about the role of individuals responsible for the modification of CT acquisition protocols with the same answer options. Multiple roles could be selected and were analyzed as non-exclusive categories (see ESM Table 3). The additional option to state ‘The protocol cannot be modified once established’ was included to reflect situations, particularly in research and trial settings, where this can be mandatory. The answer options regarding the frequency of protocol updates included: no updates, updates as needed, less than yearly, yearly, or more than yearly.

### Technical factors

The first technical question asked whether automatic exposure control (AEC) was used. Subsequent items focused on technical parameters, including detector configuration, reconstruction algorithm type (filtered-back projection, iterative reconstruction (IR) with statistical modeling or deep learning support), reconstruction kernel, slice thickness, and interval between slices. For survey responses that included two different reconstructed slice thicknesses for a single device, only the smaller slice thickness was included in the analysis, under the pragmatic assumption (not directly surveyed) that thicker reconstructed slices were used for visualization purposes only. In the final question, respondents were asked to indicate which software functions were utilized for screening, specifically for nodule detection, nodule volumetry, calculation of VDT, aid in diagnosis (decision-support tools beyond detection/measurement [16]), structured reporting or none.

### Survey distribution and promotion

Survey participants were institutional representatives involved in LCS within the SOLACE consortium and its affiliated networks. Eligibility was defined as institutions implementing LCS protocols. Participation was voluntary. This was a retrospective international survey of anonymized professional responses; no patient data were collected, and according to institutional and national regulations, formal ethics committee approval and written informed consent were not required. The survey adhered to ESR ethical standards and GDPR regulations for data protection and privacy. It was administered internally within the SOLACE consortium from June to November 2024 and broadly disseminated from March to September 2025, closing on September 15, 2025. Reopening of the survey is planned for 2028 to provide a timely update, see Konrad et al [4] and https://redcap.link/CT_Protocol_LCS. The survey included the optional provision of an email address for follow-up. The survey was designed for completion in between 5 and 10 min, depending on the number of devices and protocols reported. Invitations were sent directly to institutional leaders within the SOLACE consortium and the Lung Cancer Policy Network. Additional promotion was supported by ESTI, EIBALL, and ALA. Dissemination messages included a survey link.

### Data analysis

Analyses were predefined as purely descriptive, and no inferential or hypothesis‑testing procedures were planned. Survey responses were automatically collected and managed using REDCap electronic data capture tools hosted at Heidelberg University Hospital [17]. Descriptive statistics were calculated and visualized using R version 4.1.3 [18]. All available responses were included on a per‑item basis, leading to varying denominators across questions.

## Results

The survey elicited 273 response attempts. Responses containing sufficient partial data were received from 71 institutions from 29 countries (see Table 1), of which 38 completed the full survey. The number of used CT devices varied between institutions. Only one CT device was used by 36, two devices were used by 18, and more than two devices were used by 17 institutions. A more detailed list of the used devices can be found in Table 2. Of the overall 140 devices described, 23 device names appeared only once in the survey, while the names of high-end devices appeared more frequently in relation to the manufacturers, e.g., Aquilion Prime SP (3), Revolution Apex (5), Spectral CT 7500 (3), Naeotom Alpha (11). The list of device names only includes device names that refer to known commercial products, resulting in 43 different device names listed. Ambiguous terms have been excluded.

**Table 1:** Countries (number of screening facilities that provided survey answers if more than one) in which survey respondents’ lung cancer screening facilities were located

Country	
Argentina	Ireland
Australia	Italy (6)
Belgium	Israel (2)
Bulgaria	Kuwait
Cameroon	Mexico
Canada (2)	Netherlands
China (2)	Norway
Croatia (4)	Poland (2)
Czechia (3)	Portugal (3)
Denmark (2)	Russia
Estonia	Spain (5)
France (2)	Switzerland (3)
Germany (6)	United Kingdom (4)
Greece (2)	United States of America (8)
Hungary (3)

**Table 2:** CT devices used in the survey respondents’ lung cancer screening facilities

Manufacturer	Number of devices	Device names, alphabetically sorted (number)
Canon	12	Aquilion 64 (2), Aquilion 80, Aquilion Lightning, Aquilion One, Aquilion One 320, Aquilion One Genesis, Aquilion Prime SP (3)
GE	38	CT VCT, Discovery CT750 HD, Lightspeed VCT 64 (2), Optima 520, Optima 660 (2), Revolution 256 (2), Revolution Apex (5), Revolution Ascend, Revolution CT (5), Revolution Evo, Revolution Frontier, Revolution GSI, Revolution HD, Revolution Maxima
Philips	10	Access CT, CT5300, iCT256, Incisive CT, IQon (2), Spectral CT 7500 (3)
Siemens	69	Definition (4), Definition AS (3), Definition AS+ (2), Edge (3), Flash (4), Force (9), Drive (5), go.All, go.Top (9), go.Up, Naeotom Alpha (11), Sensation 64, X.cite (2), X.ceed (3)
United Imaging	2	uCT530, uCT760
NA	9	(Not specified)

The number of devices and the sum of provided names differ because not every device name was given alongside the manufacturer. Quantities of device names are indicated in parentheses. All Siemens devices belong to the SOMATOM series, except for the Naeotom Alpha. Ambiguous device name entries were omitted

*NA* not available (as the manufacturer’s name was not provided)

### Institutional factors

**Fig. 1:**
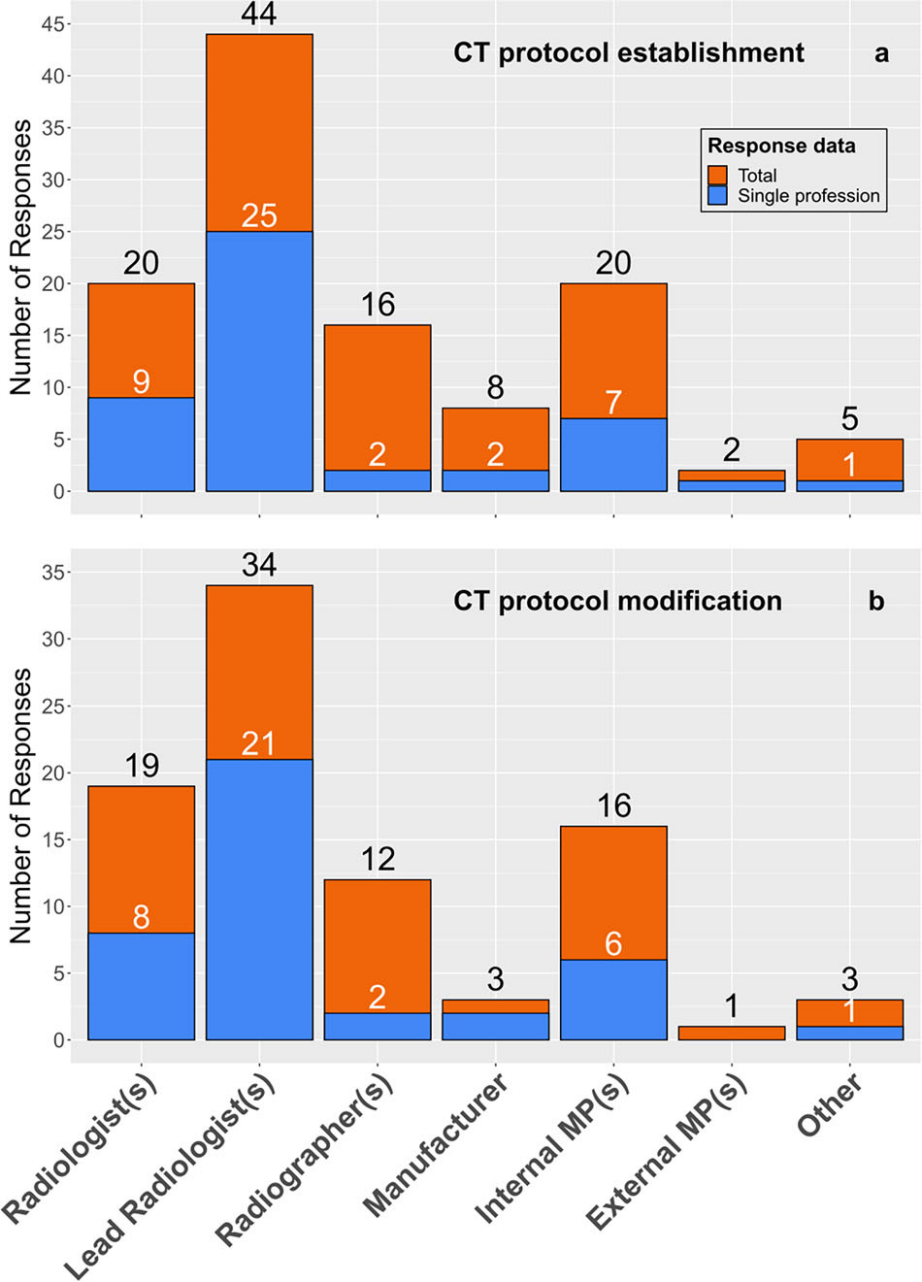
Professional roles responsible for the establishment (**a**) and modification (**b**) of the applied CT image acquisition protocols in lung cancer screening facilities. Total response data includes responses from institutions with single- and multiprofessional establishment/modification, while single profession explicitly includes answers from institutions where only one profession was responsible. MP, medical physicist

The survey answers regarding protocol establishment in Fig. 1a show that with 64 of 115, the professions responsible were radiologists in more than 50% of the cases (44 lead radiologist(s), 20 radiologist(s), respectively). Many institutions (47 of 71, 66%) had only a single profession responsible for protocol establishment, and in that case, this was primarily radiologists as well (34 of 47, 72%). The answers shown in Fig. 1b indicate that the distribution of professions responsible for the modification of protocols was similar to the establishment; however, 11 institutions were omitted, as they stated that no modification could be applied. Again, for the modification of protocols, only one professional group was responsible in 40 out of 60 (66%) institutions.

Other roles responsible for protocol establishment included the screening program director (2), a pulmonologist (1), and two protocols were defined in national or scientific programs.

Other roles responsible for protocol modification included the screening program director (1), a pulmonologist (1), and a biomedical engineer (1).

**Fig. 2:**
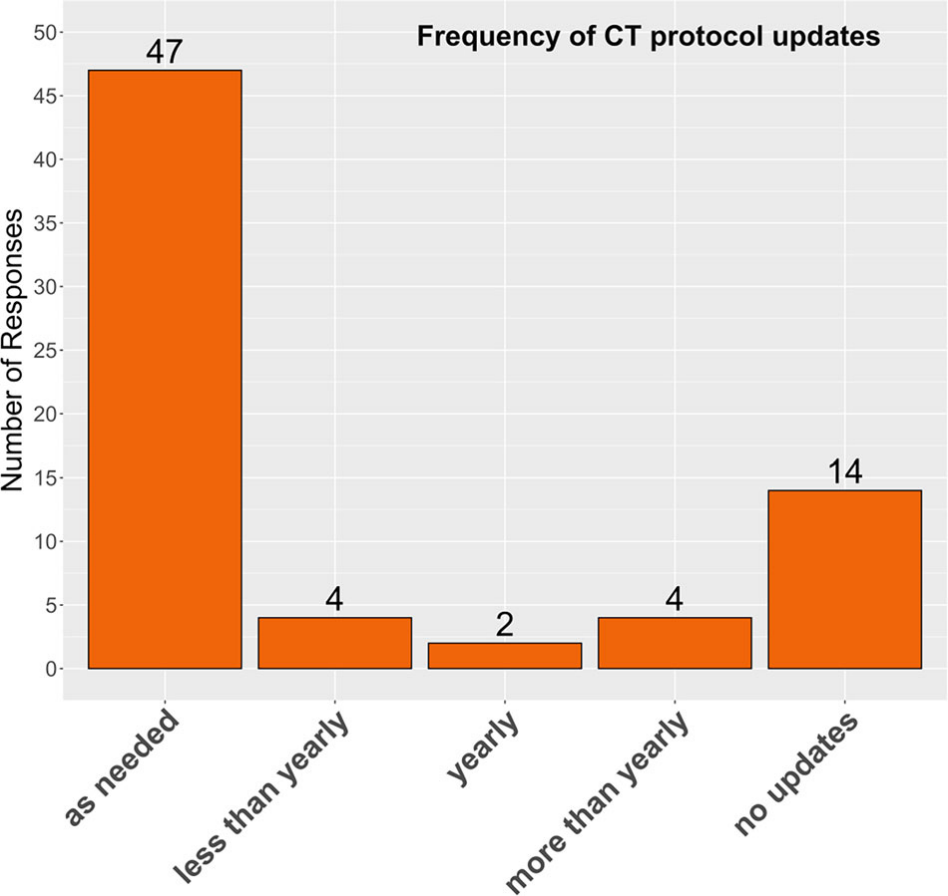
Frequency of CT image acquisition protocol updates per lung cancer screening facility

With 66% (47 of 71), the majority of protocol update frequencies stated was ‘as needed’ (Fig. 2). Here, there were 14 instances where ‘no updates’ was indicated, which implies that no updates were planned in three institutions, although updates would be applicable (since only 11 institutions stated that the protocol cannot be modified after establishment).

### Technical factors

**Fig. 3:**
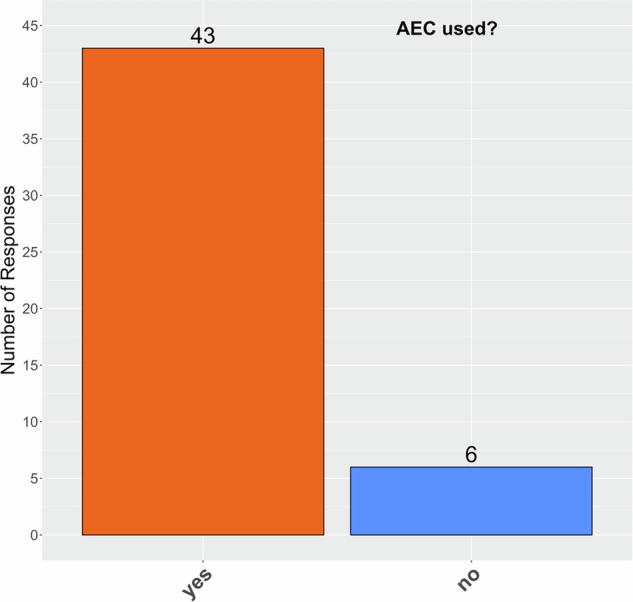
Declared use of automatic exposure control (AEC) in CT image acquisition protocols for lung cancer screening

**Fig. 4:**
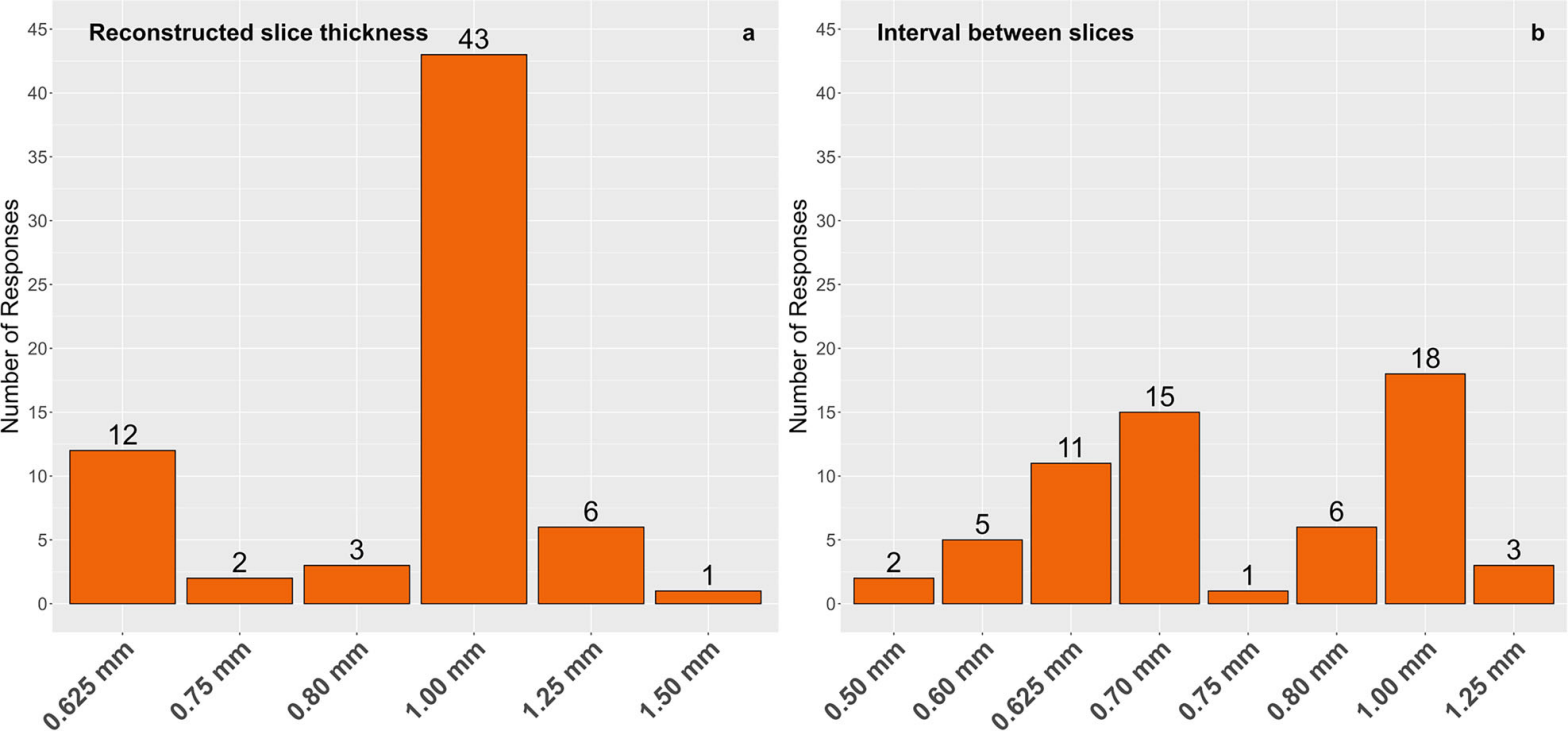
Slice thickness and interval in CT image acquisition protocols in lung cancer screening. Applied reconstructed slice thickness (**a**) and the interval between slices (**b**)

The use of AEC in LCS was confirmed for 88% of the institutions (Fig. 3). Survey responses to the topic of detector configuration demonstrated considerable heterogeneity in understanding, with some answers indicating misconceptions or divergent interpretations relative to the intended question (see verbatim answers in ESM Table 1—expected answer: Number of detector rows × Detector width mm). The used reconstruction kernels varied greatly, and survey answers are listed verbatim in ESM Table 2. The reconstructed slice thickness in CT images ranged between 0.625 and 1.50 mm. Of the 67 responses, 64% (43 of 67) included the use of 1.00 mm slice thickness (Fig. 4a). The distance between two consecutive reconstructed images (‘interval’ between slices) ranged between 0.50 mm and 1.25 mm and featured a wider array of variants than the slice thickness (Fig. 4b). As this interval is a post-processing parameter in image reconstruction, it does not depend on the physical details of the detector geometry. The interval was generally less than or equal to the reconstructed slice thickness and therefore guaranteed a contiguous or overlapping implementation.

**Fig. 5:**
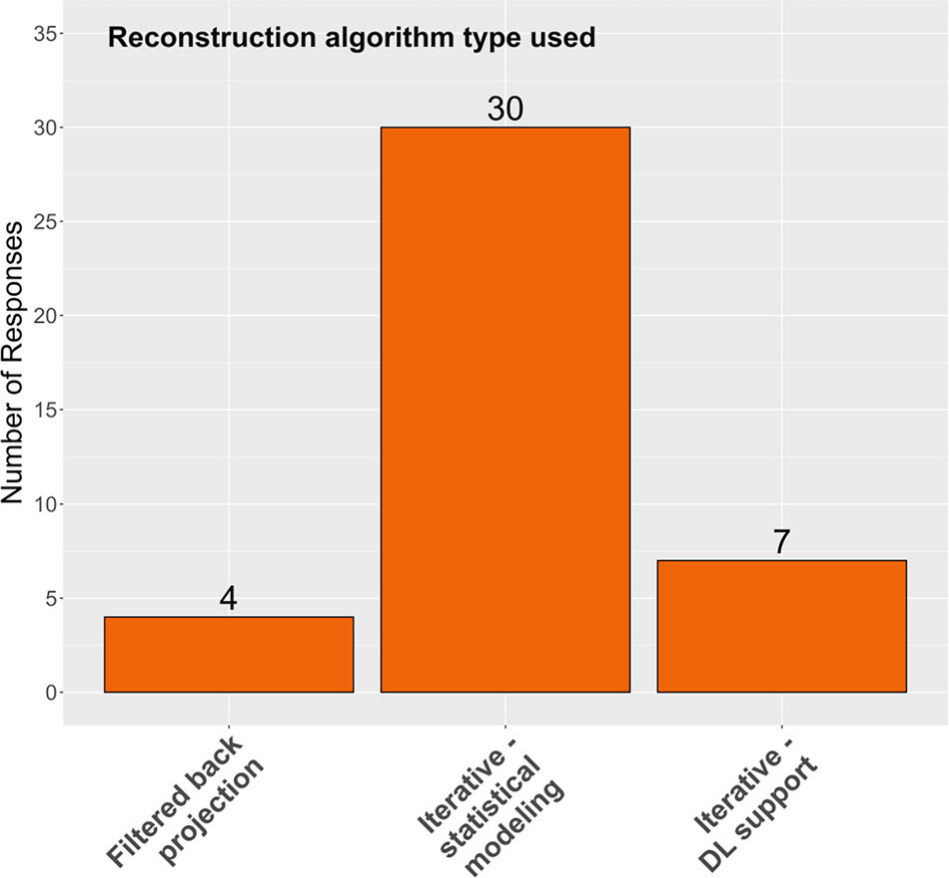
Applied reconstruction algorithm types in CT image acquisition protocols in lung cancer screening. DL, deep learning

The distribution of reconstruction algorithm types used for image reconstruction is presented in Fig. 5 and included filtered-back projection (4), IR with statistical modeling (30), and IR with deep learning support (7).

**Fig. 6:**
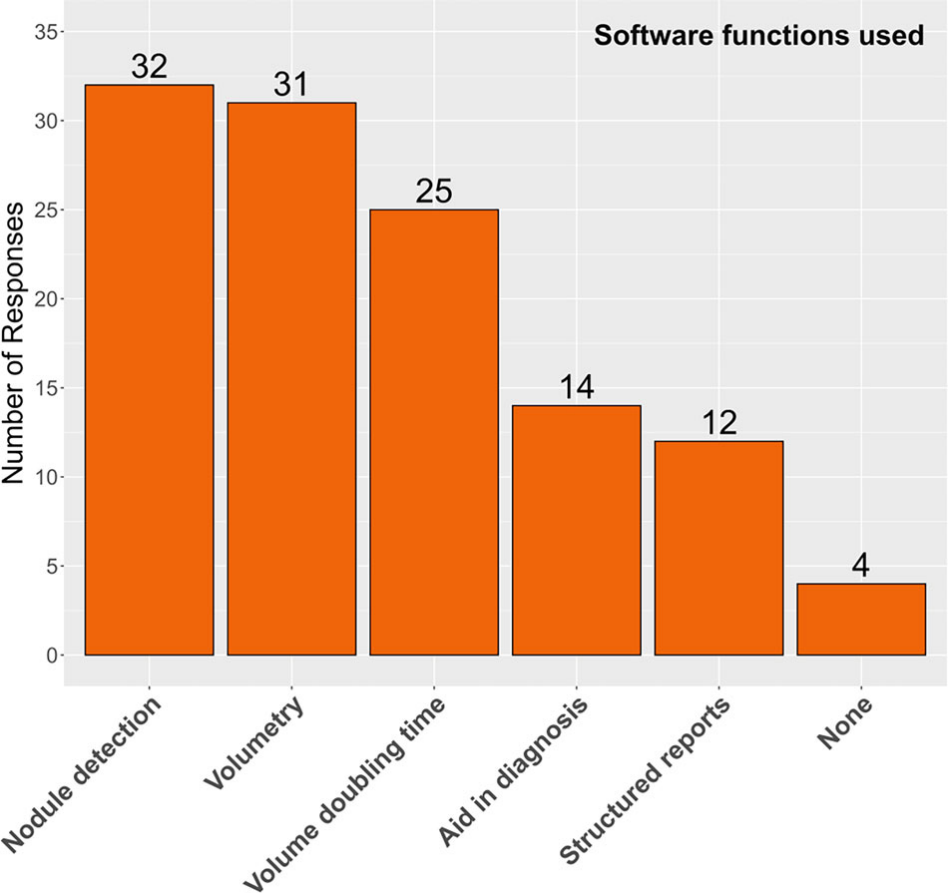
Applied software functions to support lung cancer screening in the survey respondents’ screening facilities

The distribution of the software functions used for support of LCS processes is presented in Fig. 6. The use of software functions for support of LCS was confirmed by 90% (35 of 39) of respondents answering this survey question. The majority of these 35 respondents used software functions for nodule detection (92%), the volumetry of nodules (89%), and the calculation of the VDT (71%). Only 40% used software for aid in diagnosis, and 34% used software for structured reports. These percentages reflect reported utilization and do not provide information on software performance or clinical effectiveness.

## Discussion

This international survey reveals pronounced diversity in LCS practices, likely shaped by differences in national regulatory frameworks and financial circumstances. Despite the known benefits of a multiprofessional approach in protocol development, only a third of the responding institutions reported involving more than one professional group in establishing CT acquisition protocols, and the same was true for protocol modifications. Protocol update frequency remains inconsistent, with no standardized schedule identified.

The survey results demonstrate substantial heterogeneity in the types and manufacturers of CT devices employed for LCS, reflecting the varied technical landscape across participating institutions. Notably, there was confusion among respondents regarding detector configuration, indicating potential misunderstandings of key technical parameters. This highlights the need for improved clarity regarding key technical terminology.

Most institutions reported implementing AEC, indicating widespread trust in automation processes for radiation dose reduction. Reconstructed slice thicknesses were generally at or below 1.0 mm, and image intervals matched or were smaller than the reconstructed thickness. This practice assures contiguous or overlapping CT image slices, which is optimal for nodule detection and volumetry. However, considerable variability was reported in the reconstruction kernels used, underscoring the need for greater harmonization.

The survey also captured a shift away from conventional filtered-back projection toward IR, including a growing number of institutions adopting deep learning IR. While this transition may offer advantages in image quality and dose reduction, it is important to recognize that deep learning IR can alter image characteristics depending on the applied reconstruction kernel. As a result, the reconstructed images may not fully represent the physical reality of lung nodules. The subsequent application of AI-based software for nodule detection, volumetry, and VDT calculation layered on these reconstructed images could potentially amplify discrepancies. However, given the lack of neutral, comprehensive validation studies on the integration of advanced reconstruction algorithms with AI analytic tools, these concerns remain largely theoretical and warrant further investigation. Cautious interpretation of such combined approaches is therefore advisable until robust evidence addressing their accuracy and reliability is available.

The observed heterogeneity in CT acquisition parameters and protocol governance identified in the survey aligns with longstanding evidence on the substantial influence of image reconstruction parameters on the accuracy and reproducibility of lung nodule volumetry. Studies such as Honda et al demonstrated the impact of reconstruction kernel and slice thickness on volumetry precision, highlighting the need for standardized protocols [19]. This survey’s findings of variable reconstruction kernel usage and occasional misunderstandings in detector configuration echo these concerns. The observed misconceptions regarding detector configuration suggest that some technical concepts are not consistently understood across centers, which may also affect the reliability of other detailed technical responses and highlight a need for targeted education and clearer protocol documentation. These findings underline that LCS protocol definition and maintenance should be a genuinely multi‑professional task including medical physicists and radiographers, as also highlighted in recent local QA recommendations [20].

The diversity of CT devices from different manufacturers mirrors the dynamic evolution of CT technology, including advances in spectral imaging and dose reduction [21, 22]. Such heterogeneity complicates efforts toward protocol harmonization. Consistent QA remains paramount; the National Lung Screening Trial demonstrated the role of rigorous QA in improving biomarker reliability in multicenter trials [23]. This survey’s findings of inconsistent multiprofessional involvement in establishing and updating protocols reflect potential QA gaps. Recent guidance from the QMIC Small Lung Nodule Biomarker Committee emphasizes phantom-based validation and standardized imaging parameters for improving reproducibility [24, 25].

Examples from UK screening trials showcase the critical role of phantom-validated, standardized scanning protocols in optimizing image quality and radiation dose [26]. The ESTI LCS technical standards represent concerted efforts toward harmonization, but our results reveal incomplete alignment with these guidelines [27].

US recommendations, notably from the American Association of Physicists in Medicine (AAPM) [28], differ by allowing somewhat higher slice thicknesses and radiation doses, influenced by differing regulatory frameworks. For example, German legislation enforces strict low-dose CT criteria in LCS [11]. These jurisdictional realities affect protocol adoption and underscore the complexity of developing universally applicable standards.

The survey reflects not only regional variation but also a broad, global interest in protocol standardization, as demonstrated by geographically diverse responses from multiple countries. This calls attention to the critical role of cross-continental collaborations in driving harmonized best practices while respecting national legal and resource constraints [11, 20, 27, 28].

Precise nodule risk stratification benefits from volume-based thresholds to predict malignancy more accurately than diameter alone, supporting the importance of reproducible volumetry [29]. Yet, knowledge gaps remain regarding volumetry software performance across varied protocols [30, 31].

Shifts in image acquisition toward the latest devices promise consistent diagnostic quality and are crucial for expanding screening to detect comorbidities like emphysema and coronary artery calcification [22, 32, 33]. In particular, it is expected that radiation exposure during CT scans will be minimized to a level close to that of chest x-rays. These multiparametric capabilities challenge protocol design but also highlight the necessity for flexible, standardized imaging frameworks.

Study limitations to consider are: First, the respondent sample likely represents primarily professionals responsible for protocol decisions in research-oriented LCS institutions, as evidenced by the frequent use of high-end CT devices. This potentially limits generalizability to other practice settings. Second, response rates to individual survey items varied; technical questions in particular often went unanswered, possibly reflecting either a lack of technical background or lower perceived relevance among some respondents. Third, while the absolute number of responses appears modest, this is reasonable when referenced against the relatively small pool of potential respondents, namely, those directly overseeing CT protocols at active LCS sites, a role frequently held by a single professional group per institution.

Additional limitations include possible selection bias due to survey distribution channels favoring academic or consortium-affiliated centers and the exclusion of sites not performing established LCS, which may underrepresent protocol diversity globally. The survey was not formally validated and did not define a core item set, so partial responses may introduce missingness bias that cannot be fully characterized. Moreover, the survey did not distinguish CT systems dedicated exclusively to screening from those shared with diagnostic workflows, which may influence protocol stability and technical consistency. Reliance on self-reporting introduces potential for misinterpretation or recall bias, particularly for complex technical details. Some technical items, including software functions, were formulated broadly and may have been interpreted heterogeneously by respondents. Despite these constraints, the findings complement trial-based recommendations such as those by Lancaster et al [32] and provide a valuable snapshot of current international practices and highlight areas for future research and harmonization.

In conclusion, the survey data highlight critical challenges and opportunities aligning with current scientific understanding and evolving technical recommendations. Harmonizing protocols, enhancing multidisciplinary engagement in protocol governance, and embedding rigorous QA practices are paramount to optimizing LCS efficacy across diverse healthcare environments.

## Supplementary information

ELECTRONIC SUPPLEMENTARY MATERIAL

## Data Availability

The survey data are stored in the secure REDCap database hosted by Heidelberg University Hospital. Access is restricted to authorized study personnel in accordance with institutional policies and GDPR regulations. The dataset may contain potentially identifiable or personal information of survey respondents and is therefore not publicly available.

## Abbreviations

AEC: Automatic exposure control
ALA: American Lung Association
EIBALL: European Imaging Biomarkers Alliance
ERS: European Respiratory Society
ESM: Electronic Supplementary Material
ESR: European Society of Radiology
ESTI: European Society of Thoracic Imaging
IR: Iterative reconstruction
LCS: Lung cancer screening
QA: Quality assurance
QMIC: Quantitative Medical Imaging Coalition
SOLACE: Strengthening the screening of lung cancer in Europe
VDT: Volume doubling time
