# Supplementary material for: CT acquisition protocols in lung cancer screening: implications for guideline development from a worldwide survey
Source: Insights Imaging. 2026 Mar 16;17:70. doi: 10.1186/s13244-026-02239-y (PMC12992829; doi:10.1186/s13244-026-02239-y)
Supplement: Supplementary file 1 — ELECTRONIC SUPPLEMENTARY MATERIAL [file 13244_2026_2239_MOESM1_ESM.pdf]

CT acquisition protocols in lung cancer screening: implications for guideline development from a worldwide survey

Electronic Supplementary Material

ESM Table 1 Verbatim answers provided in the CT acquisition protocol survey from lung cancer screening facilities for the survey request ‘Please indicate the detector collimation settings (detector configurations) used in the LCS CT acquisition protocol(s)’

**Collimation settings (detector configuration)**	
128x0.6	0.6
40	Auto 128x0,625
80 mm, pitch 0.99	128 × 0.6, 40 × 0.6, 64 × 0.6
SN100	80mm
40mm	unsure
0.8 mm	0.6 mm
0.75	LCS detector
1.0 thickness, 0.7 increment	0,7
0.625	128 x 0.6 mm
0.6	64x0.6mm
128x0.6 (DRIVE), 64 x 0.625 (Rev HD), 32 x 0.7 (Go.All)	1.25
64 x 0.6 mm	32
3 mm	128x0.6
1.25 or less (smallest available)	64x0.6
kV120, eff. mAs 7	64x1.5
80 x 0.5mm	192x0.6
64*0.625	38.4mm
0.2 mm	GE Revolution 128x0.625 Siemens Somatom x.Cite 64x0.6
256, 64	128x0.6mm
0.625	64x0.6mm
128	

ESM Table 2 Verbatim answers provided in the CT acquisition protocol survey when asked for device-specific ‘Kernel/Recon Type/Reconstruction Filter Name(s)’ applied in lung cancer screening facilities

**Reconstruction kernel**	
BR44 and Bl60	Br44
LUNG/MEDIASTINUM	Medium-sharp kernel (Br49)
Standard kernel + DLIR-M recon + Lung filter	Br48 S3 1mm Lung / Sa36 S3 1 mm Lung (volumetry) / Sa36 S3 3 mm Mediastinum
Br 36 and br64	60/lung/standard
Standard Recon Type/none image enhance filter/enhanced contrast off	I31f, I70f kernel, iterative reconstruction, filter 3
Bone, Helical Plus, ASIR-V 50%; Stnd, Helical plus, DLIR High	Simens old software - B70F lung and B31f mediastinum Siemens new software - Br 60 lung and Br 40 mediastinum
bl 64 q3	BR36 S3 and BR64 S3
BI 56	Bf37 and Br59
Soft	LUNG/MEDIASTINUM
Standard soft tissue	Bone, Helical Plus, ASIR-V 50-70%
ASIR V 80	bl 64 q3
Bf39	B60
Br40/ Qr60/ BI56 S3	Bl37Admire3
I50f	Standard soft tissue
Varies mediastinal and lung	Standard
Bl64, Br36	B64
FC5 for lung for volumetry, FC18 for soft tissue	Varies mediastinal and lung
Pi lung standar and pi soft tissue smooth	B+Y/A
Bl 56	B_SHARP_C, KARL 3D 5/9
B+Y/A	Standard lung
B60, B45	FC18
Br40	Kernel Body lung special 57
IMR/SharpPlus/Lung	GE - lung and mediastinum kernel Unitedimaging - 5 lung, 0 mediastinum
Bl60, ADMIRE 3/5	LUNG/MEDIASTINUM
Standard lung	Standard soft tissue

ESM Table 3 Full survey regarding CT acquisition protocols applied in lung cancer screening facilities

Survey questions	Answer options
Country of screening centre	<drop down list>
Institution name (confidential)	<text field>
Email address (optional, confidential)	<text field>
Is one device dedicated for screening or are multiple devices used?	One Device
(Single-answer question)	Two Devices
	More than two devices
Device Name:	<text field>
Manufacturer Name:	<text field>
**Institutional factors**	
What was the role of the individual(s) who established the acquisition protocol?	Lead radiologist(s)
(Selection of all answers possible)	Radiologist(s)
	Radiographer(s)
	Manufacturer
	Internal medical physicist(s)
	External medical physicist(s)
	Other
What is the role of the individual(s) who modifies the acquisition protocol?	The protocol cannot be modified once established.
(Selection of all answers possible)	Lead radiologist(s)
	Radiologist(s)
	Radiographer(s)
	Manufacturer
	Internal medical physicist(s)
	External medical physicist(s)
	Other
What is the frequency of protocol updates?	No updates are made.

Survey questions	Answer options
(Single-answer question)	As needed
	Less than yearly
	Yearly
	More than yearly
**Technical factors**	
Is automatic exposure control (AEC) used for dose modulation?	Yes
(Single-answer question)	No
Please indicate the detector collimation settings (detector configurations) used in the LCS CT acquisition protocol(s).	<text field>
Please indicate the reconstruction algorithm type used.	Filtered back projection
(Single-answer question)	Iterative algorithm with statistical modeling
	Iterative algorithm with deep learning support
Please provide the following information for the <device name>:	
Kernel/Recon Type/Reconstruction Filter Name(s)	<text field>
Reconstructed Slice Thickness (mm)	<text field>
Increment/Reconstruction Interval (mm)	<text field>
Please indicate which software functions are used for screening:	Software for nodule detection
(Selection of all answers possible)	Software for nodule volumetry
	Software for calculation of volume doubling time
	Software for aid in diagnosis
	Software for structured reports
	None
